# A therapeutic chimeric IgG/IgA expressed by CHO cells for oral treatment of PED in piglets

**DOI:** 10.3389/fmicb.2022.1018748

**Published:** 2022-10-03

**Authors:** Yan Xiao, Yunjing Zhang, Zhiyan Wang, Wenyin Zhao, Xin Xu, Xiao Chen, Feifei Tan, Zhe Sun, Baicheng Huang, Kegong Tian

**Affiliations:** ^1^College of Veterinary Medicine, Henan Agricultural University, Zhengzhou, Henan, China; ^2^National Research Center for Veterinary Medicine, Luoyang, Henan, China; ^3^Research Center for Intelligent Computing Platforms, Zhejiang Laboratory, Hangzhou, China

**Keywords:** porcine epidemic diarrhea virus, immunoglobulin A, Chinese hamster ovary cell, cell line, oral treatment

## Abstract

Immunoglobulin A (IgA) of sows is critically important for assessing piglets’ protective capacity against porcine epidemic diarrhea virus (PEDV). Here, we report a therapeutic chimeric anti-PEDV IgG/IgA expressed by Chinese hamster ovary (CHO) cells for oral treatment of PED. The chimeric anti-PEDV IgG/IgA was produced by the CHO cell lines, in which the heavy chain was constructed by combining the VH, Cγ1 and hinge regions of PEDV IgG mAb 8A3, and the Cα2 and Cα3 domains of a Mus musculus immunoglobulin alpha chain. The chimeric anti-PEDV IgG/IgA could neutralize the strains of CV777 (G1), P014 (G2) and HN1303 (G2) *in vitro* effectively, showing broad-spectrum neutralization activity. The *in vivo* challenge experiments demonstrated that chimeric anti-PEDV IgG/IgA (9C4) produced in the CHO cell supernatant could alleviate clinical diarrhea symptoms of the PEDV infection in piglets. In general, our study showed that chimeric anti-PEDV IgG/IgA produced from CHO cell line supernatants effectively alleviates PEDV infection in piglets, which also gives the foundation for the construction of fully functional secretory IgA by the J chain introduction to maximize the antibody therapeutic effect.

## Introduction

Porcine epidemic diarrhea virus (PEDV) was first reported in Europe in the early 1970s as the strain CV777 (Pensaert and de Bouck, 1978), which is the viral pathogen caused PED in suckling piglets with severe diarrhea, vomiting and dehydration. PEDV has caused severe epidemics since the 1990s in some Asia countries, such as in Japan (Kusanagi et al., 1992) and Korea (Choi et al., 2014). The devastating outbreaks of PEDV in China since 2006 (Chen et al., 2010; Li et al., 2012; Yang et al., 2013) and in the United States since 2013 (Stevenson et al., 2013; Vlasova et al., 2014) led to a severe threat to swine health. Until now, PEDV was still the primary viral pathogen causing porcine diarrhea in China. The mortality rate of suckling piglets caused by PED often reaches 80–100%, resulting in severe economic losses to the swine industry.

PEDV is an enveloped, positive-sense, single-stranded RNA virus belonging to the genus *Alphacoronavirus* in the family Coronaviridae, order Nidovirales. Among the four PEDV structural proteins, namely spike (S), envelope (E), membrane (M), and nucleocapsid (N), the S protein dominates the surface of virus particles and mediates direct binding to cell receptors, making it a significant target to induce neutralizing antibodies (Chang et al., 2002; Wicht et al., 2014).

PEDV IgA and IgG neutralizing antibodies in serum, colostrum and milk, especially secretory IgA (sIgA), from late-term pregnant sows after PEDV vaccination play an essential role in protecting piglets from PEDV infection (Chattha et al., 2015; Ouyang et al., 2015). The sIgA in maternal colostrum and milk can be directly transmitted to piglets in the form of lactation, limiting the replication of PEDV in the intestinal tract and protecting piglets against clinical disease by direct virus neutralization (Zimmerman et al., 2019). Therefore, for PEDV, oral administration of antibodies like IgA may have an immediate therapeutic effect.

In eukaryotic cell culture systems, the Chinese Hamster Ovary (CHO) cells are mainly used for the mass-production of the high-quality therapeutic recombinant proteins and monoclonal antibodies (mAbs; Sharker and Rahman, 2021), such as the spike ectodomain of SARS-CoV-2 (Djaileb et al., 2021), a MERS-coronavirus vaccine antigen (Nyon et al., 2018), the mAbs of structurally unstable G protein-coupled receptor CC chemokine receptor 9 (Nanamiya et al., 2021), the immunoglobulin new antigen receptor of cartilaginous fishes (Enatsu et al., 2021), and functional chimeric sIgA (Berdoz et al., 1999).

To achieve the *in vitro* expression of anti-PEDV IgA, we constructed a CHO cell line stably expressing the chimeric anti-PEDV IgG/IgA antibody. The biological activities of the chimeric anti-PEDV IgG/IgA were characterized, and the therapeutic effect in pigs was evaluated.

## Materials and methods

### Animals

Piglets (*n* = 15) of 15-day-old used in this study were tested PEDV negative by RT-PCR (primers: forward, 5’-TATGGCTTGCATCACTCTTA-3′; reverse, 5′– TTGACTGAACGACCAACACG-3′), and also tested negative of transmissible gastroenteritis virus, porcine delta-coronavirus, and porcine rotavirus in RT-PCR assay. Animal experiments were conducted at National Research Center for Veterinary Medicine for treatment efficacy studies. The experimental protocols were approved by the Institutional Animal Care and Use Committee of the National Research Center for Veterinary Medicine (Permit Number: 20211101055).

### Virus

Three PEDV strains of different genotypes were used in this study, the classical G1 strain CV777 (GenBank: KU664503), G2 strains P014 and HN1303 (GenBank: KR080551). The propagation of PEDV was performed as described previously (Wang et al., 2015). Briefly, the monolayer of Vero cells was prepared in Dulbecco’s modified Eagle’s medium (DMEM) supplemented with 5% fetal bovine serum (HyClone, Logan, UT, United States) at 37°C in a 5% CO_2_ incubator for 24 h. The PEDV infected cells were cultured in the medium containing 2.5 μg/ml trypsin (Sigma-Aldrich, MO, United States) and 3% tryptose phosphate broth solution (Sigma-Aldrich). The virus was harvested when the cells showed an 80% cytopathic effect (CPE) and stored at −80°C until use.

### Construction of chimeric IgG/IgA vector

**Figure 1:**
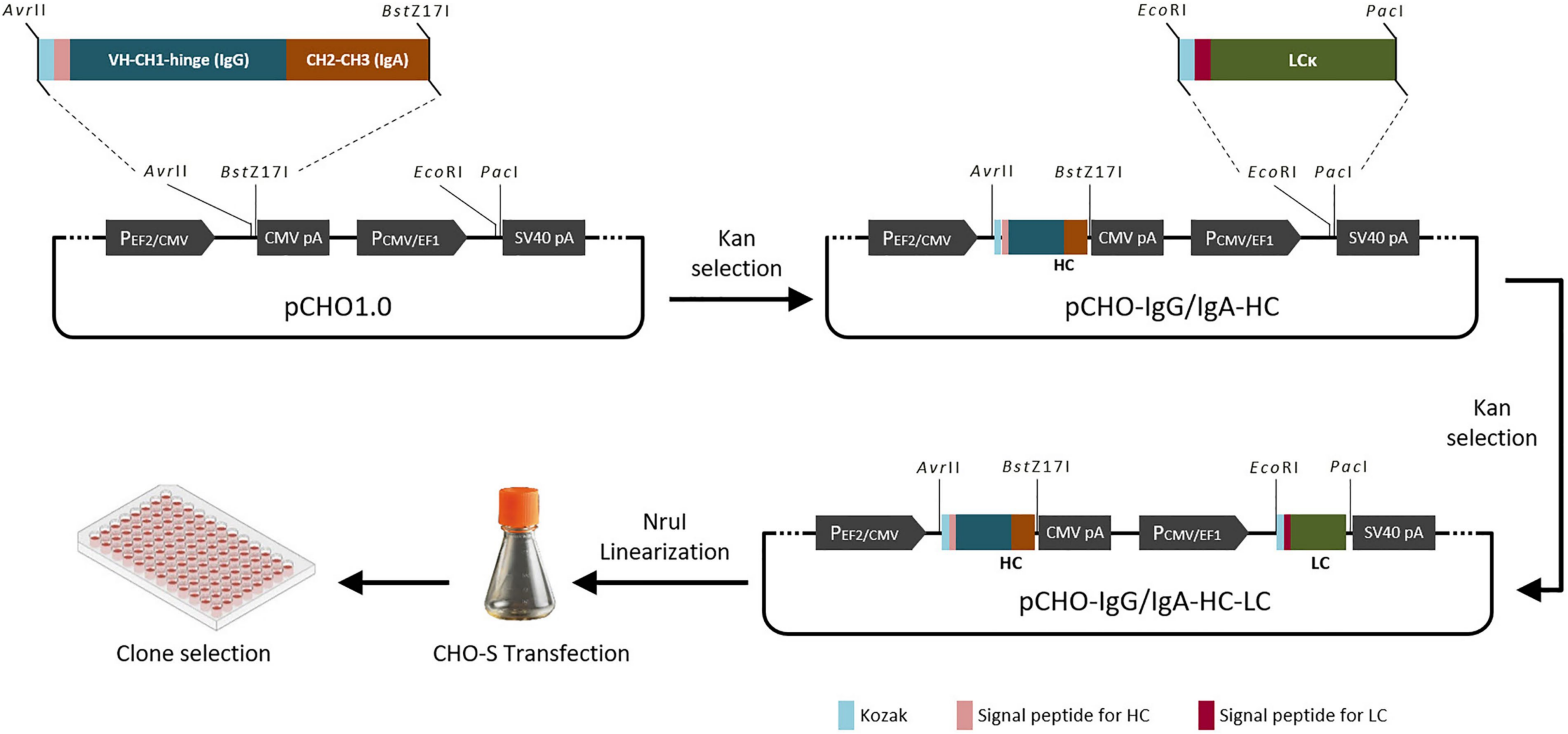
Schematic diagram of chimeric antibody construction.

The structure of the chimeric anti-PED IgG/IgA heavy chain gene was designed according to the strategy described previously (Tobisawa et al., 2011). The heavy chain of the chimeric IgG/IgA consists of the VH, Cγ1 and hinge regions from a PEDV IgG mAb 8A3 (Zhang et al., 2016), and the Cα2 and Cα3 domains from a *Mus musculus* immunoglobulin alpha chain (GenBank accession number: AB644393). In the case of the light chain, the mAb 8A3 was selected for the chimeric anti-PED IgG/IgA construction. The antibody DNA fragments were synthesized commercially (Genewiz, China) with CHO codon optimization. Thus, the chimeric anti-PED IgG/IgA vectors based on pCHO1.0 (Thermo Fisher Scientific, United States) for CHO cell transfection were constructed using the heavy and light chain genes prepared above, which were amplified from the synthesized DNA using the following primers: heavy chain, *Avr*II-HC-For (5’-TCCTAGGgccaccatggactggacctg-3′) and *Bst*Z17I-HC-Rev (5’-CGTATACTTAGTAGCAGATTCCATCTCC-3′); light chain, *EcoR*V-LC-For (5’-TTGATATCgccaccatggcctggatgat-3′) and *Pac*I-LC-Rev (5’-CGTTAATTAACGT CAACACTCATTCC-3′). The fragment of the heavy chain was inserted into the pCHO1.0 plasmid by digesting with *Avr* II and *BstZ* 17I, and then the ligation product was transformed into competent cells for the positive clone selection of pCHO-IgG/IgA-HC. After that, the light chain fragment amplified by *EcoR*V-LC-For and *Pac*I-LC-Rev was inserted into the plasmid pCHO-IgG/IgA-HC after digestion with *EcoR* V and *Pac* I and then be transformed into competent cells for the positive clone selection of pCHO-IgG/IgA-HC-LC. Finally, the plasmid pCHO-IgG/IgA-HC-LC harboring genes of heavy and light chains was confirmed by sequencing. The construction process is shown in Figure 1.

### Cell line selection

CHO-S cells (Thermo Fisher Scientific, United States) were maintained in complete CD FortiCHO Medium (Gibco, United States) in a 37°C incubator containing a humidified atmosphere of 8% CO_2_ in an orbital shaker platform (Infors HT, Switzerland) rotating at 120 revolutions per minute. The transfection of pCHO-8A3-IgG/IgA was according to the kit instructions. Briefly, on the day of transfection, the CHO-S cells were seeded in 125-ml polycarbonate Erlenmeyer flasks (Corning, United States) with 1.0 × 10^6^ cells/mL in 30 ml of complete CD FortiCHO Medium, and then the cells were transfected with the *Nru* I linearized pCHO-8A3-IgG/IgA (0.5 μg) that mixed with FreeStyle™ MAX Reagent and OptiPRO SFM. At 48 h after transfection, the selection of stable transfectants was proceeded in two phases by adding the MTX (Sigma Aldrich, A6770) and puromycin according to the instructions. The positive clones were identified by a specific dot blot. After that, the high-yield clones proceeded to a series of cell passages. The concentration of chimeric IgG/IgA was detected by IgA Mouse ELISA Kit (Invitrogen). The recombinant chimeric anti-PED IgG/IgA in the supernatant were analyzed by sodium dodecyl sulfate polyacrylamide gel electrophoresis (SDS-PAGE).

### SDS–PAGE and western blot

SDS–PAGE and immunoblotting were performed under reducing and non-reducing conditions (Lu et al., 2013). In western blot assay, IgG/IgA was detected by the 1:2000 diluted HRP-conjugated goat anti-mouse IgG (H + L) and Anti-Mouse IgA (α-chain specific) − Peroxidase antibody produced in goat (Sigma, United States). The signals representing α-heavy and light chains were enzymatically detected using a chemiluminescence reagent (Pierce, United States).

### Neutralization test of PEDV mAbs

Vero cells were seeded in 96-well plates (3 × 10^4^ cell/well) at 37°C for 24 h. PEDV viral stock was diluted with DMEM (10 μg/ml trypsin, 0.3% tryptose phosphate broth) to 2000 TCID_50_/mL. The mAbs (8A3 purified from mouse ascites, 2 mg/ml; 9C4, 15F6 and 20E5, CHO supernatant) were 2-fold serial diluted (8A3, 1:8–1:512; 9C4, 15F6 and 20E5: 1:2–1:128) with DMEM (10 μg/ml trypsin, 0.3% tryptose phosphate broth) for an equal volume (250 μl, each) mixing with PEDV viral solution prepared above, and then incubate at 37°C for 1 h. Mixtures of mAbs and viruses were added to the cells (100 μl/well, quadruplicate), and then the plates were incubated at 37°C with 5% CO_2_ for 3–5 days for CPE observation. Neutralizing endpoint titers were defined as the highest mAb dilution preventing CPE occurrence. Serum samples with neutralizing endpoint titers ≥1:20 were considered positive for PEDV-neutralizing antibodies (Poonsuk et al., 2016).

### Challenge and oral treatment

Fifteen piglets (15-day-old) were equally divided into three groups. Group 1 was the unchallenged control, and groups 2 and 3 were orally challenged with strain HN1303 at the dose of 1.0 ml (10^3^ TCID_50_/mL) per piglet. Group 2 was designed for oral treatment of chimeric anti-PED IgG/IgA in the cell culture supernatant. Group 3 was the infected control (treatment with culture medium). After the appearance of typical diarrhea symptoms, in a phase of 3 days, the piglets in group 2 were given the supernatant containing chimeric anti-PED IgG/IgA orally 10 ml (~ 0.5 mg/ml) per piglet each time and six times daily, and the piglets in group 3 were given the culture medium following the procedure as group 2. The symptoms of piglets were recorded for evaluation of treatment effect. Small intestine samples of jejunum were collected for immunohistochemistry (IHC) assay.

### Detection of fecal viral load

PEDV viral RNA in fecal samples was tested according to the reported method (Kao et al., 2018). Briefly, rectal swabs were resuspended in PBS, and the RNA was extracted from 200 μl of stool suspension. After cDNA reverse-transcription, it was used for the analysis of quantitative real-time PCR (qPCR). qPCR was conducted using the previously reported primer-probe (Jung et al., 2014).

### Immunohistochemistry

Immunohistochemistry (IHC) assay was carried out according to the reported method (Wang et al., 2015). Briefly, small intestine samples were de-waxed with xylene, rehydrated by graded alcohols, and then air-dried. Antigen retrieval was accomplished with 0.01 mol/l citrate buffer (pH 6.0) boiling for 15 min, followed by 3 PBS rinses. The slides were next incubated at 4°C for 12 h with PEDV mAb 3F12 (diluted 1:500). After three washes with PBS, sections were flooded and incubated for 1 h at 37°C with 1:100 diluted HRP-goat anti-mouse IgG (Biomedical Technologies, United States). After being washed three times in PBS, the slides were incubated for 5 min at room temperature in Diaminobenzidine solution (ZSGB-BIO, Beijing, China).

### Statistical analysis

The data of neutralization ability (100%) were presented as mean ± SD. The differences between the three PEDV strains in chimeric-IgG/IgA neutralization ability were determined using Student’s t-test, and fecal viral loading were analyzed statistically with Two-way ANOVA. Differences were considered statistically significant when *p* < 0.05.

## Results

### Construction of the mammalian plasmid for chimeric IgG/IgA expression

To obtain rearranged immunoglobulin genes of PEDV-specific IgG, mRNA of hybridoma clone 8A3A10 was isolated (Zhang et al., 2016). After transformation, the positive colonies with heavy and light chains of 8A3 were sent for sequencing. The sequencing result showed that the heavy chain of 8A3A10 was IgG2a and the light chain type of κ. The heavy and light chains of the chimeric IgG/IgA were synthesized to obtain the plasmid for CHO transfection. The chimeric-IgG/IgA heavy chain DNA was amplified by PCR using the IgA-AvrII-For and IgA-BstZ17I-Rev primer set, and the light chain DNA was amplified by PCR using the EcoRV-For and PacI-Rev primer set. Firstly, the target DNA of the heavy chain was ligated into the pCHO1.0 vector between the *Avr* II and *BstZ* 17I, and then the target DNA of the light chain was ligated into the pCHO1.0 with the heavy chain insertion. The DNA sequences of the positive clones in each step were confirmed by sequencing.

### The construction of cell lines expressing chimeric IgG/IgA

**Figure 2:**
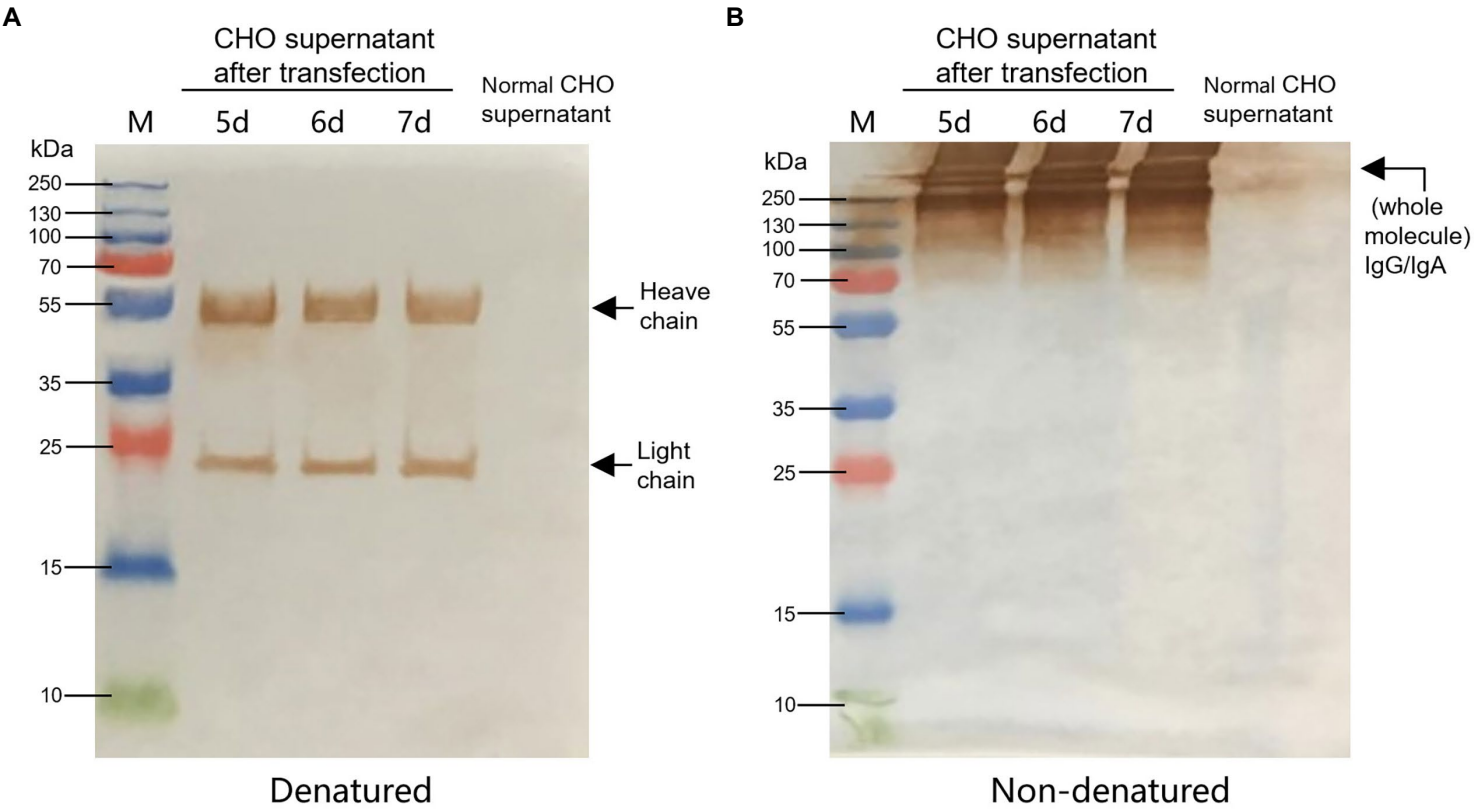
Identification of CHO-expressed chimeric IgG/IgA. Western blot analysis of CHO-expressed chimeric IgG/IgA under reducing conditions **(A)** and non-reducing conditions **(B)** in the 5, 6 and 7 dpt. Black arrows indicated the heavy and light chains.

**Figure 3:**
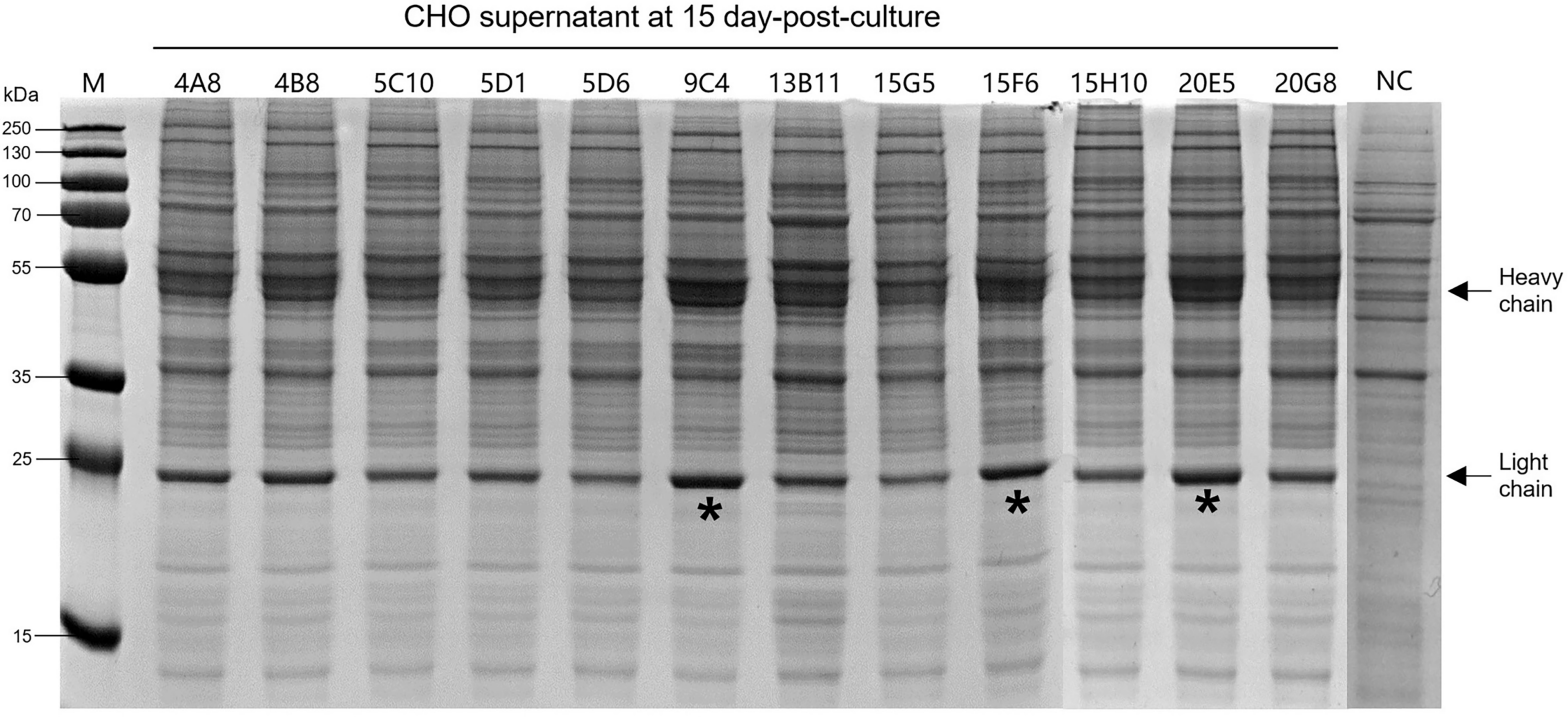
Expression levels of the 12 selected CHO clones after 15-day incubation. Black arrows indicated the heavy and light chains. The “*” indicated three clones with higher expression levels. NC, normal cell of CHO.

According to the instructions, the chimeric-IgG/IgA heavy and IgG light (κ) constructs were simultaneously transfected into CHO cells, and transient expression of the dimeric chimeric-IgG/IgA was attempted. Recombinant dimeric IgA was also produced as control by expressing the IgA heavy and IgA-associated light. The result of transient expression showed a correct expression of the dimeric hybrid-IgG/IgA (Supplementary Figure 1; Figure 2). After two-phase selection, 68 clones were selected by dot WB (Supplementary Figure 2), then 12 clones with high expression levels were selected for the SDS-PAGE assay. As expected, the bands of the IgG/IgA heavy and IgG light were detected in the SDS-PAGE assay (Figure 3). The chimeric IgG/IgA amount showed the highest accumulation at 15 day-post-culture in the CHO cell line, approximately 0.5 mg/ml tested by IgA Mouse ELISA Kit.

### Broad neutralization of chimeric IgG/IgA

**Figure 4:**
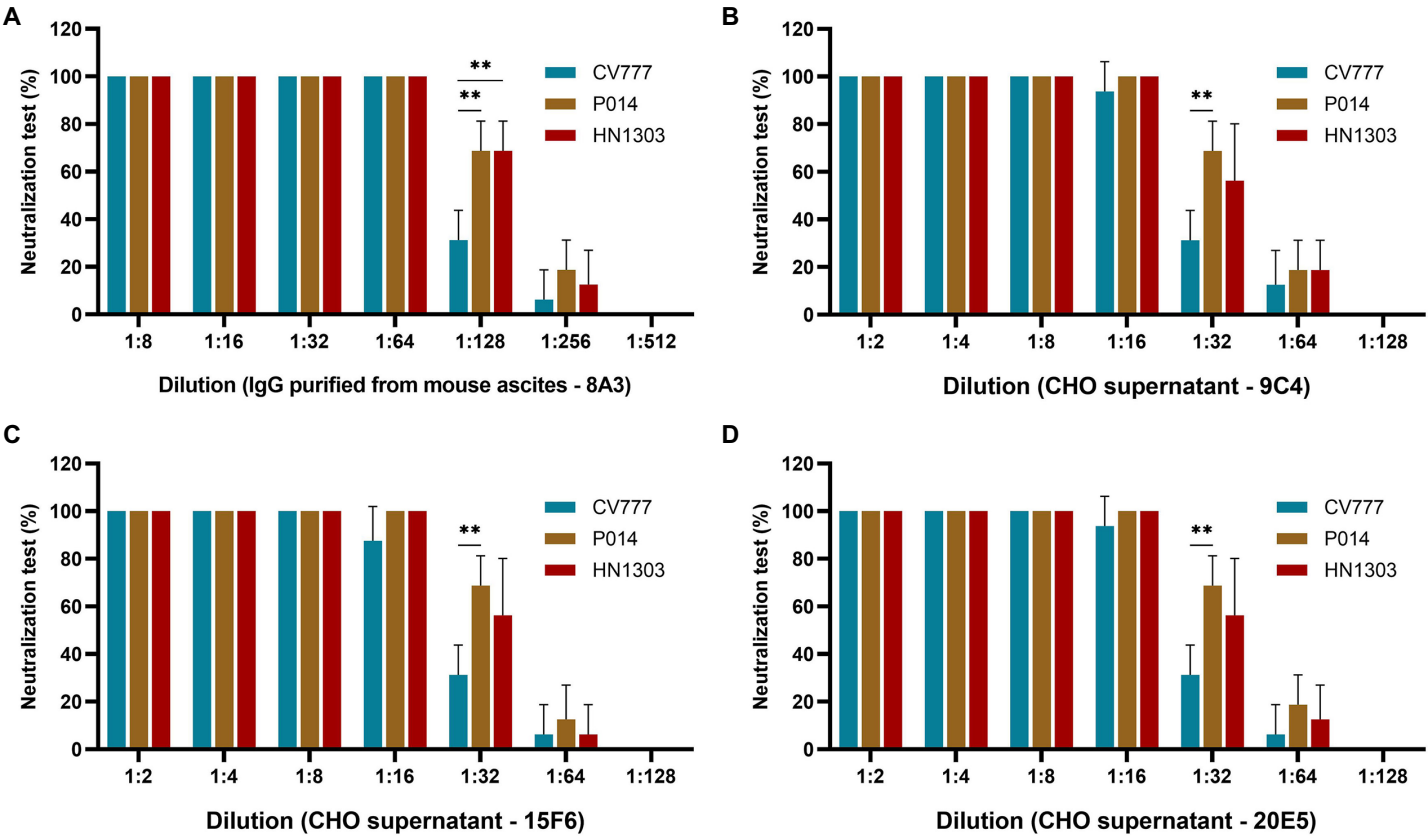
Evaluation of the neutralization effect of chimeric IgG/IgA in different genotypes of PEDV strains. The neutralization assay results of purified IgG 8A3 **(A)**, CHO cell supernatant of 9C4 **(B)**, 15F6 **(C)** and 20E5 **(D)** against PEDV strains CV777, P014 and HN1303. **p < 0.01.

After the SDS-PAGE assay, cell supernatants of 3 clones (9C4, 15F6, and 20E5) were collected for neutralization assay. As shown in Figure 4, the cell supernatants from the three clones showed no significant difference in neutralization ability to the PEDV strains of P014 (G2a) and HN1303 (G2b), while all showed a slight lower neutralization ability to CV777 (G1), which may be caused by the reason of 8A3 is a G2 strain-specific mAb. After that, the clone 9C4 was selected for the therapeutic efficacy study for its highest yield (Figure 3).

### Therapeutic efficacy studies of chimeric IgG/IgA

**Figure 5:**
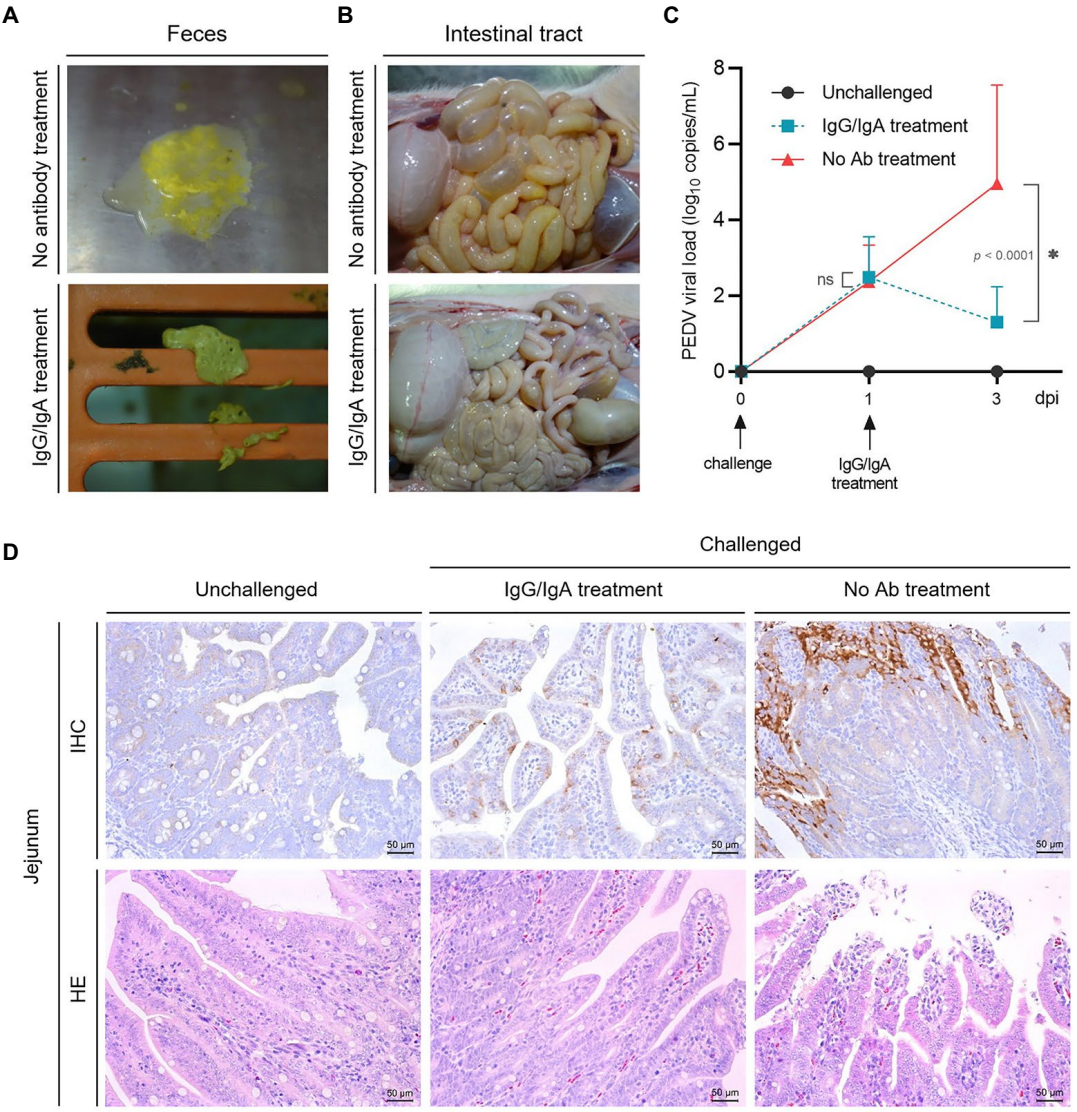
Evaluation of the therapeutic effect of chimeric IgG/IgA produced by CHO cell line. Piglets feces **(A)** and intestinal tract **(B)** in group 2 (one day after IgG/IgA treatment) and 3 (no antibody treatment). **(C)** The PEDV RNA copies in the feces of piglets, the error bars indicated standard deviation. Data were analyzed using two-way ANOVA by time. **(D)** IHC and HE assay of the piglets in group 1 (unchallenged), 2 (IgG/IgA treatment) and 3 (no antibody treatment). dpi, day-post-infection.

At 24–36 h-post-challenge (hpc), piglets in groups 2 and 3 showed typical clinical symptoms like poor appetite and softening of feces, and then the piglets in group 2 were oral treatment as designed immediately. The results showed that 4 of 5 piglets in group 2 showed significant recovery from 24 h after oral treatment with chimeric anti-PED IgG/IgA (in the cell culture supernatant, six times a day), such as the normal appetite and dry solid feces (Figure 5A), while all the piglets in group 3 continue to show the clinical symptoms until 48–120 hpc, and five piglets died in 96–120 hpc. The piglets in group 1 showed no abnormalities throughout the experimental trial.

Anatomically, the intestinal tissues of one selected recovered piglet from group 2 showed a much lighter intestinal lesions than piglet from group 3 (Figure 5B). From the results of viral shedding in stool, the PEDV viral load of the IgG/IgA treatment group was significantly lower than that of the No Ab treatment group after antibody treatment (Figure 5C), although the viral load detection of stool samples had a larger standard deviation value. Pathologically, compared with the piglet from group 3, the small intestinal tissues of piglet from group 2 showed a much lighter intestinal villi damage (Figure 5D). As well, a smaller area of positive antigen distribution in the jejunum could be found in piglets from group 2 than in group 3 in the IHC assay (Figure 5D).

## Discussion

For enteric viruses, vaccination to induce lactogenic immunity and their transmission to suckling neonates *via* colostrum and milk are pivotal for early passive protection (Chattha et al., 2015). IgA, especially sIgA, in mammary secretions of sows is critically important for assessing the protective capacity against PEDV for piglets (Mazanec et al., 1992; Suzuki et al., 2015; Langel et al., 2016).

The major neutralizing epitopes of PEDV locates on S protein, which would be easily lost in conventional virus concentration methods such as sucrose gradient centrifugation and leads to the low sensitivity of the ELISA method (Ko et al., 2009). Due to the high variability of S protein, the traditional vaccines may provide only limited protection (Sun et al., 2012). Thus, other effective ways are needed to control the occurrence of the disease. Therefore, this study aims to achieve the eukaryotic expression of a chimeric IgG/IgA with high specificity to PEDV.

In this study, PEDV MAb 8A3 was selected for chimeric IgG/IgA expression using the CHO expression system, which would likely ensure the native conformation of the sites recognized by the MAbs. We constructed the CHO cell line with stable expression of the chimeric IgG/IgA, which has more industrial advantages than antibodies produced by transient transfection (Shi et al., 2021). Moreover, the expression of chimeric IgG/IgA here would give the foundation for the construction of fully functional sIgA, this requires the introduction of the J chain into the chimeric IgG/IgA (Tobisawa et al., 2011) in the future.

The neutralization activity of the chimeric IgG/IgA expressed in the CHO cell line was tested in this study against PEDV G1 and G2 strains. We found that the three CHO clones with chimeric IgG/IgA expression showed no significant difference in neutralization titer among the three strains. This result demonstrates that the chimeric IgG/IgA expressed in the CHO cell line has broad-spectrum neutralization activity, providing the strategy to address the limitations of conventional vaccines.

Due to the presence of proteases in the gastrointestinal tract, the absorbable IgA in colostrum and sow’s milk is reduced, and only a limited IgA can reach the intestinal tract for virus neutralization. Thus, oral supplementation of highly neutralizing active antibodies could serve as an effective strategy for passive immunization to directly neutralize pathogens in the animal’s gut (Moor et al., 2017). Here, the piglets, which had not ingested colostrum to exclude the influence of maternally-derived neutralizing antibodies, orally treated with chimeric IgG/IgA successfully alleviated clinical diarrhea symptoms after the PEDV challenge, we believe this is due to an increase of effective antibodies that reached the gut by active feeding.

Since we are in the experimental model of treatment after the piglet showed symptoms, we use a larger amount of antibodies in the treatment design, and the relief of the symptoms indicates that the antibody we expressed has a sufficient therapeutic effect, it is more demonstrative in the antibody intervention by post-onset strategy than a preventive treatment modality in PED control (Shi et al., 2021). Undoubtedly, in practice, the amount of PEDV chimeric IgG/IgA produced by the CHO cell line is more suitable for large-scale clinical application.

Because we found the expression level of chimeric IgG/IgA was significantly lower than that of recombinant IgG, our future research directions for PEDV chimeric IgG/IgA are to optimize the production process in the CHO cell line and delivery method in the application, to maximize the antibody therapeutic effect.

## Data availability statement

The original contributions presented in the study are included in the article/Supplementary material, further inquiries can be directed to the corresponding authors.

## Ethics statement

All applicable international, national, and/or institutional guidelines for the care and use of animals were followed. This study was carried out by using pigs for antibody evaluation and efficacy studies. Ethical approval of collecting samples from pigs was obtained by the Research Ethics Committee of the National Research Center for Veterinary Medicine in 2021.

## Author contributions

YX, BH, and KT conceived and designed the research. BH, YZ, ZW, WZ, XX, and ZS conducted the experiments. YX, FT, and BH analyzed the data. BH and KT wrote the manuscript. All the authors read and approved the manuscript.

## Funding

This study was supported by the grant of R&D and industrialization of genetically engineered vaccines for swine pseudorabies, swine ring and *Mycoplasma hyopneumoniae* (201200211200).

## Conflict of interest

The authors declare that the research was conducted in the absence of any commercial or financial relationships that could be construed as a potential conflict of interest.

## Publisher’s note

All claims expressed in this article are solely those of the authors and do not necessarily represent those of their affiliated organizations, or those of the publisher, the editors and the reviewers. Any product that may be evaluated in this article, or claim that may be made by its manufacturer, is not guaranteed or endorsed by the publisher.

## Supplementary material

SUPPLEMENTARY FIGURE 1 Identification of CHO-expressed chimeric IgG/IgA by SDS-PAGE. SDS-PAGE analysis of CHO-expressed chimeric IgG/IgA under reducing conditions **(A)** and non-reducing conditions **(B)** in the 5, 6 and 7 day-post-transfection (dpt). Black arrows indicated the heavy and light chains.

SUPPLEMENTARY FIGURE 2 Identification of the PEDV chimeric IgG/IgA expression level in CHO clones. Red circles indicate 12 CHO clones selected for IgG/IgA expression quantification. The positive and negative control dots in the WB assay are located at the bottom right of the membrane.
