# Supplementary figures and images for: A therapeutic chimeric IgG/IgA expressed by CHO cells for oral treatment of PED in piglets
Source: Front Microbiol. 2022 Oct 3;13:1018748. doi: 10.3389/fmicb.2022.1018748 (PMC9574063; doi:10.3389/fmicb.2022.1018748)

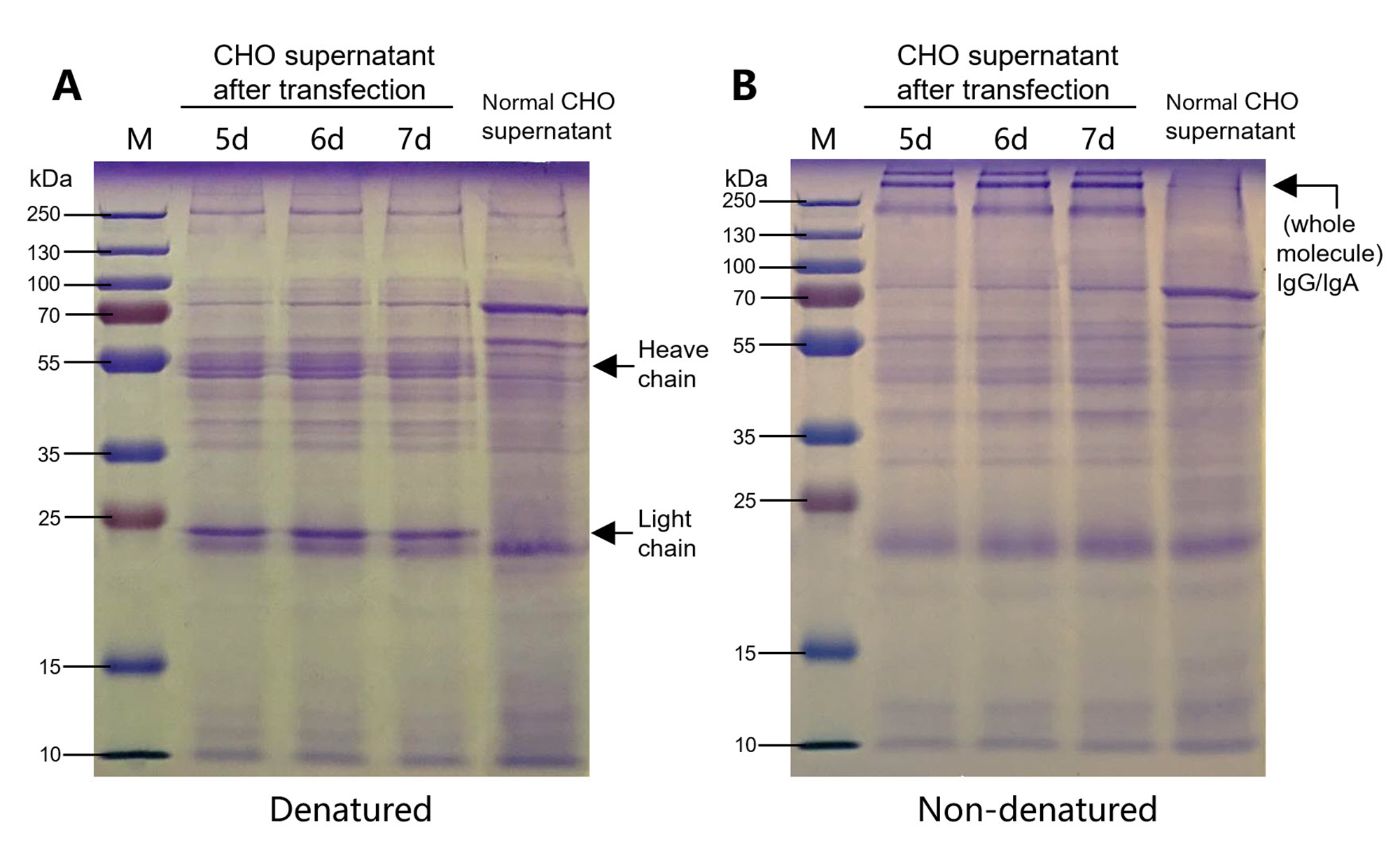

Supplement: SUPPLEMENTARY FIGURE 1 — Identification of CHO-expressed chimeric IgG/IgA by SDS-PAGE. SDS-PAGE analysis of CHO-expressed chimeric IgG/IgA under reducing conditions (A) and non-reducing conditions (B) in the 5, 6 and 7 day-post-transfection (dpt). Black arrows indicated the heavy and light chains. [file Image_1.JPEG]

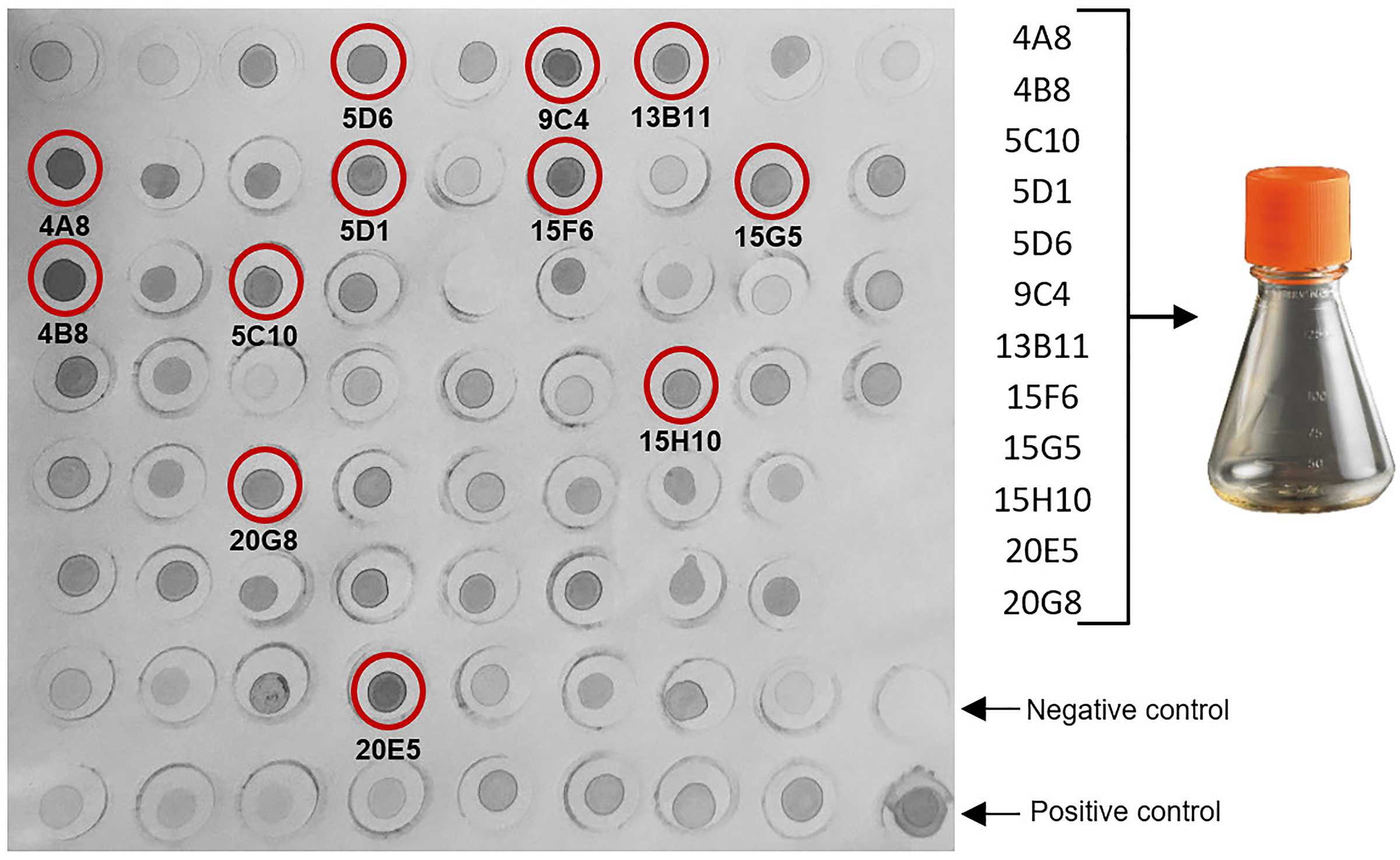

Supplement: SUPPLEMENTARY FIGURE 2 — Identification of the PEDV chimeric IgG/IgA expression level in CHO clones. Red circles indicate 12 CHO clones selected for IgG/IgA expression quantification. The positive and negative control dots in the WB assay are located at the bottom right of the membrane. [file Image_2.JPEG]
